# Distinct *Schistosoma mansoni*-Specific Immunoglobulin Subclasses Are Induced by Different *Schistosoma mansoni* Stages—A Tool to Decipher *Schistosoma mansoni* Infection Stages

**DOI:** 10.3390/pathogens11010019

**Published:** 2021-12-24

**Authors:** Kathrin Arndts, Tayseer E. M. Elfaki, Michael J. Doenhoff, Gnatoulma Katawa, Ibtisam A. Goreish, Misk El Yemen A. Atti El Mekki, Achim Hoerauf, Manuel Ritter, Laura E. Layland

**Affiliations:** 1 Institute of Medical Microbiology, Immunology and Parasitology (IMMIP), University Hospital Bonn (UKB), 53127 Bonn, Germany; Kathrin.arndts@ukbonn.de (K.A.); hoerauf@uni-bonn.de (A.H.); manuel.ritter@ukb.uni-bonn.de (M.R.); 2 Department of Parasitology and Medical Entomology, College of Medical Laboratory Science, Sudan University of Science and Technology, Khartoum 13311, Sudan; tayseeralfaki5@gmail.com (T.E.M.E.); miskatti@yahoo.com (M.E.Y.A.A.E.M.); 3 School of Life Sciences, University of Nottingham, University Park, Nottingham NG7 2RD, UK; mbamd2@exmail.nottingham.ac.uk; 4 Unité de Recherche en Immunologie et Immunomodulation (UR2IM), Ecole Supérieure des Techniques Biologiques et Alimentaires (ESTBA), University of Lome, Lomé BP 1515, Togo; mahkatawa@yahoo.fr; 5 Animal Resources Research Corporation, Ministry of Livestock, Fisheries and Rangelands, Khartoum 13311, Sudan; ibgoreish@yahoo.com; 6 German Centre for Infection Research (DZIF), Neglected Tropical Disease, Partner Site, Bonn-Cologne, 53127 Bonn, Germany; 7 German-West African Centre for Global Health and Pandemic Prevention (G-WAC), Partner Site Bonn, 53127 Bonn, Germany

**Keywords:** *Schistosoma mansoni*, Sudan, cercarial transformation fluid (*Sm*CTF), immunoglobulins, multivariable regression analysis

## Abstract

Despite the existence of an effective medication against schistosomiasis, the disease remains a major health problem in affected areas, especially for those lacking appropriate sanitary facilities. Moreover, treatment cannot prevent re-infection since it is only effective on adult schistosome worms. Previous retrospective studies in the Sudan have discovered unique immuno-epidemiological profiles in uninfected individuals and those positive for *Schistosoma mansoni* via polymerase chain reaction (PCR) but egg-negative and those with eggs in their stool. Expanding on these data, serum samples from these individuals were further investigated for the presence of cercarial (*Sm*CTF)-specific antibodies, which would indicate immune responses at the early stages of infection. Indeed, *Sm*CTF IgG1, 2, 3 and 4 levels were significantly elevated in *Sm*PCR^+^ individuals when compared to egg^+^ patients. Following multivariable regression analysis, including *Sm*CTF-specific Igs, *Schistosoma* egg antigen (SEA)-specific and *Schistosoma* worm antigen (SWA)-specific immunoglobulins revealed a specific immunoglobulin (Ig) profile of individuals presenting different states of infection, which may be a useful future tool in order to identify egg^−^ individuals and thereby prevent unnecessary treatments.

## 1. Introduction

In a recent study from Cha et al., it was revealed that schistosomiasis continues to burden populations in non-stable communities of the Sudan [1]. In correlation with our previous studies from the Sudan [2,3], Cha and colleagues also found strong associations between infection and available latrines—since infections are obtained in fresh water sources from snails releasing cercariae, the infectious stage. Thus, despite large-scale programmes designed to tackle schistosomiasis [4], this chronic helminth infection remains a severely neglected tropical disease in the Sudan [5]. Such programmes are costly and logistically challenging, and the frequency of treatment is determined by the prevalence of infection [6]. In high transmission areas, treatment may have to be repeated every year for a number of years, adding further difficulties to ensure compliance within the communities. Cha and colleagues also revealed over 2 million people would not benefit from schistosome-specific mass drug administration (MDA) in community-wide programmes with 75% coverage, despite high endemicity (8% for schistosomiasis) [1]. Moreover, 1.7 million people would receive the drug unnecessarily, which can drive the reduction in drug susceptibility and even resistance [7]. Treatment for schistosomes remains praziquantel, an effective, quick-acting drug that kills adult worms but has no effect on earlier stages in the host and does not stop re-infections [8,9,10]. Thus, research to prevent infection requires newer approaches for alternative drugs and vaccines, and this should be coupled with limiting contact with infectious sources, such as available latrines at home, schools and washing stations [11].

Using a combination of diagnostic techniques, our earlier studies on a cohort of Sudanese individuals living in a *Schistosoma mansoni*-endemic area revealed a further subset of infected individuals that were positive for *S. mansoni* via PCR but had no detectable eggs in stool samples [2,3]. This group could be individuals with dying, menopausal or single worm infections, or an early infection. In terms of schistosome-specific antibody levels, this group showed increased levels of worm-specific IgG2 [3] but lower egg-specific IgG4 levels when compared to egg-positive individuals [2].

When free-living cercariae penetrate the mammalian host’s skin and lose their bifurcated tails in a process termed “transformation” to become schistosomulae [12,13], which linger for a few days, this elicits a local immune response in the dermis and dermal cells [14,15,16,17]. To expand on the profile of individuals presenting a *Sm*PCR^+^egg^−^ state, we determined the levels of antibodies in a *S. mansoni* cercarial antigen preparation (called cercarial transformation fluid—*Sm*CTF, described by Smith et al., 2012) [12] to identify whether the variations on “early-stage” antibodies are also different between the groups.

## 2. Results

### 2.1. All Measured SmCTF-specific IgGs Are Elevated in SmPCR^+^ Individuals

**Figure 1:**
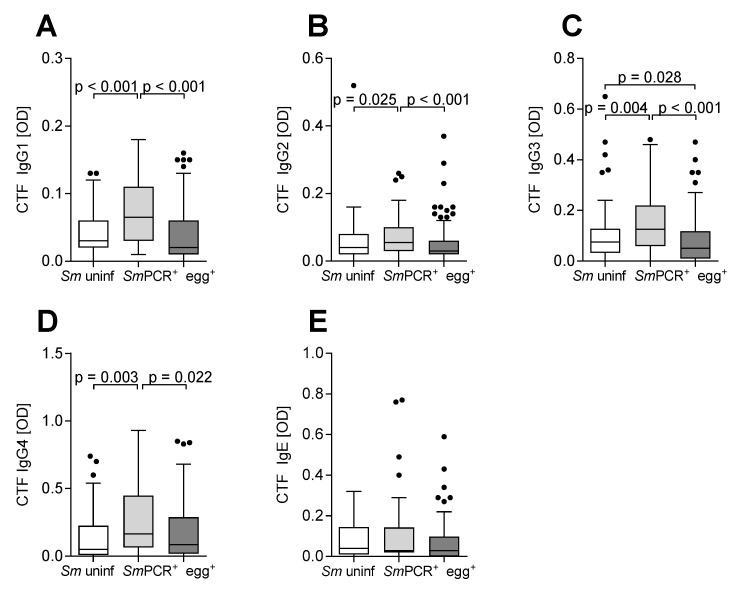
Higher levels of *Sm*CTF-specific IgGs in *Sm*PCR^+^ individuals. Serum samples from all study participants (*Sm* uninfected *n* = 60, *Sm*PCR^+^ *n* = 62, egg^+^ *n* = 108) were analysed for levels of *Sm*CTF-specific antibodies using ELISA technology. The graphs show the optical density of CTF-specific IgG1 (**A**), IgG2 (**B**), IgG3 (**C**), IgG4 (**D**) and IgE (**E**). Data are shown as box whiskers with median, interquartile ranges and outliers. Since data were non-parametric, statistical significances between the indicated groups were obtained after Kruskal–Wallis and Mann–Whitney U-tests.

Immuno-epidemiological research hypothesizes that individuals with elevated levels of anti-schistosome IgG4 are more susceptible to infection, whereas those with high anti-schistosome IgE are protected against re-infection [18,19]. Few studies however, have investigated the relationship between the parasite stage-specific antibody production and a clinical diagnostic status. Our previous studies identified a further cohort—namely *Sm*PCR^+^egg^−^ individuals [2,3]. Upon testing egg (SEA)- or worm (SWA)-specific antibody levels, we determined that both infected groups (egg^+^ and *Sm*PCR^+^) had higher SWA-specific IgG3 and IgG4, and SEA-specific IgG4, than uninfected individuals. Moreover, SWA-specific IgG2 antibody levels were higher in the *Sm*PCR^+^ group when compared to egg^+^ individuals. In experiments performed here—using extracts from cercarial life-stages—we assessed *Sm*CTF-specific Ig levels in the same cohort (Figure 1). The levels of *Sm*CTF-specific IgG1, IgG2, IgG3 and IgG4 were significantly higher in the *Sm*PCR^+^ group when compared to both uninfected and egg^+^ cohorts (Figure 1A–D). With regards to *Sm*CTF-specific IgE levels, reduced levels were observed in egg^+^ individuals when compared to both other groups, albeit not significantly (Figure 1E).

### 2.2. SmCTF IgG1 Levels Are Associated with SmPCR^+^ Individuals

The correlation analysis of egg load and levels of *Sm*CTF-specific Igs revealed a significant negative correlation with IgG1 (r = − 0.235, *p* > 0.001), IgG2 (r = − 0.151, *p* = 0.022) and IgG3 (r = − 0.265, *p* > 0.001). If the age was correlated to the *Sm*CTF-specific Igs, a significant correlation with *Sm*CTF-specific IgE (r = 0.230, *p* < 0.001) and IgG4 (r = 0.147, *p* = 0.026) was detected. An additional analysis revealed significantly higher levels of *Sm*CTF IgG1 and IgG3 in the “young”aged *Sm*PCR^+^ group when compared to “young” aged egg^+^ individuals. Several differences were also observed in the “adolescent” group since IgG1, IgG2 and IgG3 levels significantly increased in the *Sm*PCR^+^ cohort when compared to either *Sm* uninf or egg^+^ individuals. Additionally, in the “adolescent” cohort, both *Sm*PCR^+^ and egg^+^ IgG4 levels were higher than in the *Sm* uninf group. No differences were observed in the adult population (Supplementary Figure S1).

In order to include “early-stage” antibodies in our immunoprofiling of the investigated participants, we performed an initial regression analysis to determine associations of *Sm*CTF-specific Ig levels with age or data on exposure. Whereas infection *per se*, was associated with *Sm*CTF-specific IgG4 levels, there was no significant association when comparing egg^+^ individuals with uninfected participants (Table 1). In contrast, a *Sm*PCR^+^ status was more linked to elevated *Sm*CTF-specific IgG1 levels when compared to uninfected individuals. The same outcome was observed when comparing *Sm*PCR^+^ and egg^+^ individuals and, additionally, the first group was also associated with *Sm*CTF-specific IgG3 levels, whereas an egg^+^ *S. mansoni* infection was associated with *Sm*CTF-specific IgG2 levels. Associations with age groups in context with *Sm*CTF-specific Igs were consistent with our previous results; thus, younger (4–9 years old) individuals were more prone to belong to the infected groups (either *Sm*PCR^+^ or egg^+^), whereas between these two groups there was no significant association.

### 2.3. Strong Association of SEA-Specific IgG4 with Infection

**Figure 2:**
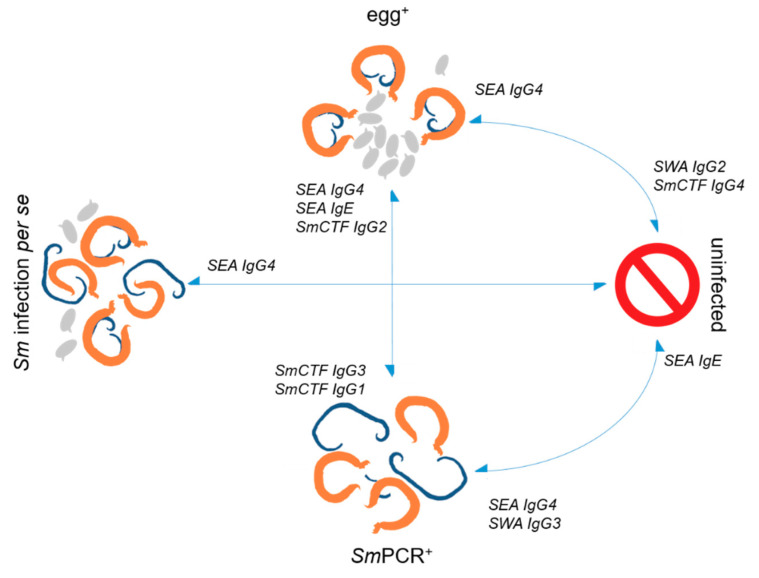
Emerging immune profiles in Sudanese individuals in endemic regions of *Schistosoma mansoni*. The *Schistosoma* worm antigen (SWA)-, soluble egg antigen (SEA) and cercarial extract-specific (*Sm*-CTF) immunoglobulins, age and exposure were compared via regression analyses between the groups, as indicated by the arrows. The *Sm*PCR^+^ group is characterised by the absence of eggs but the presence of worms. Egg^+^ individuals are classified by the presence of worms and eggs in their stool, whereas uninfected individuals lack both but live in the same endemic area. The combined “infection *per se* group” consists of egg^+^ and *Sm*PCR^+^ individuals. Indicated profiles demonstrate highly associated schistosome-specific life stage-specific IgG levels.

Next, a multivariate regression analysis was performed following a combination of all measured SEA- and SWA-specific Igs from our previous studies (Elfaki, 2016, Arndts, 2019) with our new data gathered in this study (Table 2). Interestingly, SEA-specific IgG4 remained the only schistosome-specific Ig strongly associated with infection *per se*, and SEA-specific IgE was highly associated with egg^+^ individuals when compared to *Sm*PCR^+^ individuals. This profile now includes *Sm*CTF IgG2 when compared to the *Sm*PCR^+^ cohort. In contrast, the *Sm*PCR^+^ group was more associated with *Sm*CTF-specific IgG3 and IgG1, and uninfected individuals showed associations with *Sm*CTF-specific IgG4 when compared with egg^+^ but not *Sm*PCR^+^ individuals. Thus, the picture shown in Figure 2 confirms that individuals with different infection states present different schistosome life stage-specific Igs. Thus, the analysis of schistosome stage-specific antibody levels might be a novel tool to decipher the stage of *S. mansoni* infections.

## 3. Discussion

Within this retrospective study using serum samples from a cohort from the Sudan, we could show that *Sm*CTF-specific IgG1-4 are higher in *Sm*PCR^+^ individuals when compared to egg^+^ individuals, highlighting again how schistosome-specific antibody levels change during the different infection stages. Combining all evaluated schistosome-specific Ig data sets together from previous studies with the same cohort, using multivariate regression analysis [2,3], supported these findings since egg^+^ individuals were characterised by elevated *Sm*CTF-specific IgG2, SEA-specific IgG4 and IgE levels in comparison to *Sm*PCR^+^ individuals. The latter were associated with elevated *Sm*CTF-specific IgG3 and 1 levels.

Although control programmes started more than 40 years ago in the Sudan, schistosomiasis is still endemic in all states of the country, except the Red Sea State [20], especially among the inhabitants of fragile and conflict-affected areas [21]. Assessments from the Sudanese Ministry of Health state that more than eight million people are at risk of schistosomiasis [1], and an estimated 5.8 million people (around 15% of the total population) require treatment [20]. In order to improve effective antihelminthic treatments as well as prevention of unnecessary treatment, in-depth knowledge about host–parasite interaction is needed, including schistosome-specific antibody responses [22]. The detection of anti-schistosomal antibodies is a useful addition to microscopy [23] since the latter technique is time consuming, requires expertise and has limited sensitivity in cases of low egg secretion. Moreover, antibody tests can be run in larger batches.

Initial infections by schistosomes can already occur in young children, whereas in adolescence, infections decline, implicating the development of a type of immunity that hinders re-infection [24,25,26]. Previous immuno-epidemiological studies could show that elevated levels of anti-schistosome IgG4 are associated with parasitic susceptibility, whereas anti-schistosome IgE antibodies induce resistance to re-infection [18,19]. The association between the high levels of anti-larval IgE and the resistance to a schistosoma infection was previously described [27]. However, we observed the lowest levels of *Sm*CTF-specific IgE in our infected egg^+^ group. A correlation analysis of all *Sm*CTF isotypes and age only revealed a significant, positive correlation for *Sm*CTF-specific IgE in line with the lower age within the group of egg^+^ individuals, as shown previously [2,3]. In 2013, Coulibaly et al. showed in a study performed in Cote d’Ivoire that *Sm*CTF-specific rapid diagnostic tests were comparable in terms of sensitivity when tested against Kato–Katz techniques and therefore interesting candidates for mapping the prevalence of anti-schistosome antibodies in a given population pending intervention [28]. Nausch and colleagues further confirmed these data in a study from an endemic area in Zimbabwe [29]. The detection of the early stages of schistosoma infection is favourable, especially before the egg deposition in the host tissues, as the development of severe pathology would be efficiently prevented [30]. Moreover, the cercarial antigen preparation is more easily produced than SEA [23] and was shown to perform equivalently to SEA in ELISA in the detection of anti-schistosome antibodies in sera of infected individuals [12]. Thus, the* Sm*CTF antigen may be an interesting candidate to measure serum samples of exposed individuals from endemic areas either lacking MDA, or only applying MDA after the detection of eggs in stool samples, to get a hint about potential egg-free infections that might require treatment—or the other way round—in order to prevent unnecessary treatments in children. In addition, it would be interesting to measure the functionality of such stage-specific antibodies in future studies, e.g., through co-cultivation experiments of isolated immunoglobulins and granulocytes.

Although there is a general paucity of clinical studies investigating the relationship between specific antibody production and its impact on the severity of the infection, some studies have detected increased levels of SEA-specific IgG4 in patients with severe pathology [31,32]. Even though our participants were not examined clinically, and therefore we cannot draw any conclusions about symptomatic responses, the data obtained from the combined regression analysis on the same cohort indicate that the measurement of SEA IgG4 levels might be an interesting tool in order to identify egg^−^ but infected individuals. Moreover, future studies might apply our combined panel of stage-specific Ig measurements to correlate it to different pathologic stages or to create a lateral flow test with all three antigens for an easy/non-invasive application in the field. The lack of consecutive stool sampling might be initially seen as a limitation of the study; on the other hand, it propagated this entire research project since, upon initial analysis of some parameters, we noted that the non-infected cohort had two distinct groups. However, future studies should therefore focus on molecular techniques that are field-applicable, such as LAMP assays, to detect schistosome infection in egg-free individuals.

In conclusion, the determination of combined antibody profiles (SEA-, SWA- and *Sm*CTF-specific) might allow for a more fine-tuned status of infection, e.g., if egg^−^ individuals have elevated *Sm*CTF-specific IgG1-4 levels, this would indicate a pre-patent point of infection; if they present increased SEA IgG4 levels, they might harbour old female worms that are no longer fertile any more. All of the generated data would help to prevent unnecessary treatments that are challenging in terms of logistics in remote areas and economics. For future studies in other endemic countries, using the same diagnostic parameters could determine if the stage-specific Ig-profiling revealed here is reflected in other cohorts. This would strongly substantiate both diagnostics and the respective profiling. A potential scenario to investigate these profiles could be conducted during deworming programmes.

## 4. Methods

### 4.1. Epidemiological Profiling and Serum Collection

Between March and October 2011, a survey was performed by the Ministry of Health in Kassala and Khartoum State to estimate the prevalence and intensity of schistosomiasis (*n* = 770), as already published [2,3]. Ethical clearance was approved by the Ministry of Health-Kassala State Department of Preventive Medicine Office of the anti-bilharzia and intestinal worms, New Halfa City. All individuals gave their oral informed consent, and children were only included if we received oral consent provided by a parent or legal guardian. Written consent was not necessary since the sampling procedures were always performed in the presence of a Ministry of Health representative. All individuals provided one stool sample, out of which three slides were prepared to check for the presence of parasitic eggs using the Kato–Katz technique. Within the egg^+^ group, the *S. mansoni* egg load ranged from 24 to 960 eggs/g faeces (mean = 72). In addition, urine samples were also analysed for *S. haematobium* infection. All samples were negative for *S. haematobium* eggs, *Taenia saginata* infections and for soil-transmitted helminths such as *Ascaris lumbricoides*, *Trichuris trichiura* and hookworms. For epidemiological purposes, the study participants also completed a questionnaire (e.g., health, available latrines, education etc.) on which the examiner noted their oral consent. The participants also provided a venous blood sample (approximately 5 mL), which was drawn into Vacutainer tubes (BD Biosciences Heidelberg, Germany) containing no coagulants. After five hours at room temperature, the serum was aspirated off the clot, aliquoted into cryotubes and stored at −80 °C [2,3].

### 4.2. Schistosome-Specific Diagnostics and Groupings

The presence of helminth infection was primarily diagnosed in the field within stool samples from each study participant. Using the Kato–Katz technique, 110 individuals were identified as being positive for *S. mansoni* eggs (egg^+^). No *S. haematobium* eggs were detected in urine samples [2]. A schistosome-specific PCR [2] was also performed on serum samples, and egg-negative individuals were then further divided into uninfected (*Sm* uninf, *n* = 61) and *Sm*PCR^+^ egg-negative (*n* = 63) cohorts. An overview of the entire cohort is depicted in Elfaki et al. [2].

### 4.3. Determination of SmCTF Immunoglobulins

A cercarial antigen extract of *S. mansoni* cercariae was prepared, as described in Chand et al. [23]. The levels of *Sm*CTF-specific IgG1-4 and IgE were determined using serum samples from included participants. In short, 96-well polysorb plates (Nunc, Roskilde, Denmark) were coated overnight (50 µL/well) at 4 °C with 5 µg/mL of *Sm*CTF (BioGlab Ltd., Nottingham, UK) diluted in PBS (pH 9.6). Afterwards, plates were washed three times with washing buffer (PBS, 0.05% Tween 20, pH 7.2 (Sigma-Aldrich, Munich, Germany)), followed by a washing step using 1 x PBS. Subsequently, a blocking step was then performed for one hour at room temperature (RT) using 200 µL/well of blocking buffer (PBS/1% BSA). The washing procedure was repeated, and 50 µL/well of diluted serum (1:500 for IgG1-IgG4 and 1:20 for IgE) was added to the wells. Following an overnight incubation at 4 °C, the plates were washed again, and the biotinylated secondary antibodies (IgG1: 1:1000, IgG2: 1:4000, IgG3: 1:4000, IgG4 1:15,000, all Sigma-Aldrich, IgE 1:1000, Southern Biotech, Birmingham, USA) were added to the appropriate wells (50 µL/well) for two hours at RT, followed by the wash procedure. In the next step, 50 µL/well of streptavidin-horse radish peroxidase (Roche Diagnostics, Mannheim, Germany; 1:5000) was added to each well and incubated for 45 min at room temperature. After the last washing step, 50 µL/well of substrate solution, containing tetramethylbenzidine (Sigma-Aldrich), was added to the wells. After 15 min, the reactions were stopped using 25 µL/well 2N H_2_SO_4_ (Merck KGAA, Darmstadt, Germany). The optical density was measured with a wavelength correction (450 nm and 570 nm) using a SpectraMAX ELISA reader (Molecular Devices, Sunnyvale, USA), and data were evaluated with the SOFTmax Pro 3.0 software (Molecular Devices).

### 4.4. Statistical Analysis

The data were statistically analysed using the software SPSS (IBM SPSS Statistics 22; Armonk, NY) and GraphPad PRISM version 5.02 for Windows (GraphPad Software, Inc., La Jolla, USA). The variables did not meet assumptions to allow parametric analysis; therefore, to compare the three groups, a Kruskal–Wallis test was performed and, if significant, followed by a Mann–Whitney U-test for further pairwise comparison of the groups. *p* values of less than 0.05 were considered statistically significant. For comparisons of continuous parameters, the Spearman correlation test was used. In order to determine potential indicators for the different infection statuses (‘infected versus non-infected’, ‘egg^+^ versus *Sm *uninf’, ‘*Sm*PCR^+^ versus *Sm *uninf’ and ‘*Sm*PCR^+^ versus egg^+’^), epidemiological and Ig data were assessed using two sets of a binary logistic regression model. Epidemiological covariates included age (subgrouped into “young” (4–9 years old), “adolescent” (10–19 years old), and “adult” (20–80 years old)), and “exposure” and “non exposure” (referring to individual daily water sources), as described in Elfaki et al. 2016. In the first approach, the mentioned epidemiological covariates were assessed with all *Sm*CTF immunoglobulins in a stepwise logistic regression with forward selection using thresholds of *p* < 0.1 for the entry of variables into the model. The odds ratio (OR), 95% confidence intervals (CI) and *p* values were used as estimates of the effect of each variable. In a second analysis, we combined SEA- and SWA-specific Ig data from the first two publications [2,3], with the new *Sm*CTF data measured here.

## Figures and Tables

**Table 1:** Summary levels of binary multivariable regression analysis between the different patient groups: relation of the covariates *Sm*CTF-specific Igs, age and exposure. The covariate “exposure” refers to the daily water source (e.g., village well or direct from rivers etc.). The covariate “age” was divided into “young” (4–9 years old), “adolescent” (10–19 years old) and “adult” (20–80 years old). An analysis was performed between the three different groups, and the results depict significant parameters with odds ratio (OR), confidence interval (CI) values and *p*-values. An OR above 1 refers to an association with the group that is listed first; for example, the OR of 6.571 of *Sm*CTF IgG4 in “infected versus uninfected” implies that increases in immunoglobulin (Ig) levels for one unit (optical density (OD)) signifies an association with the infected group. * Values for *Sm*CTF-specific IgG1 and 2 were low and were therefore multiplied by 100 within the regression analysis. PCR = polymerase chain reaction.

	Covariate	OR	Confidence Interval	*p* Value *
**infected versus uninfected**				
	not exposed	0.277	[0.125–0.612]	0.002
	age group			0.001
	young	5.511	[2.095–14.499]	0.001
	adolescent	3.812	[1.682–8.638]	0.001
	*Sm*CTF IgG4	6.571	[1.245–34.692]	0.027
**egg^+^ versus uninfected**				
	not exposed	0.137	[0.055–0.345]	0.000
	age group			0.002
	young	5.613	[1.995–15.794]	0.001
	adolescent	3.136	[1.362–7.220	0.007
***Sm*** **PCR^+^ versus uninfected**				
	age group			0.034
	young	4.130	[1.149–14.849]	0.030
	adolescent	3.502	[1.277–9.604]	0.015
	*Sm*CTF IgG1 × 100	1.262	[1.100–1.448]	0.001
**egg^+^ versus *Sm*PCR^+^**				
	not exposed	0.204	[0.081–0.514]	0.001
	*Sm*CTF IgG1 × 100	0.793	[0.702–0.896]	0.000
	*Sm*CTF IgG2 × 100	1.152	[1.037–1.280]	0.008
	*Sm*CTF IgG3	0.001	[0.000–0.061]	0.002

**Table 2:** A summary of binary multivariable regression analysis between the different patient groups: relation of the covariates SEA-, SWA- and *Sm*CTF-specific Igs, age and exposure. The covariate “exposure” refers to the daily water source. The covariate “age” was divided into “young” (4–9 years old), “adolescent” (10–19 years old) and “adult” (20–80 years old). Analysis was performed between the three different groups, and the results depict significant parameters with odds ratio (OR), confidence interval (CI) values and *p* -values. An OR above 1 refers to an association with the group that is listed first; for example, the OR of 14.816 of SEA IgG4 in “infected versus uninfected” implies that increases in immunoglobulin (Ig) levels for one unit (optical density (OD)) signifies an association with the infected group. * Values for SWA-specific IgG3, *Sm*CTF-specific IgG1 and 2 were low and were therefore multiplied by 100 within the regression analysis. PCR = polymerase chain reaction.

	Covariate	OR	Confidence Interval	*p* Value *
**infected versus uninfected**				
	not exposed	0.282	[0.113–0.707]	0.007
	age group			0.037
	young	3.582	[1.223–10.490]	0.020
	adolescent	2.750	[1.055–7.172]	0.039
	SEA IgG4	14.816	[5.956–36.885]	0.000
**egg^+^ versus uninfected**				
	not exposed	0.185	[0.065–0.528]	0.002
	SWA IgG2	0.170	[0.023–1.239]	0.080
	*Sm*CTF IgG4	0.019	[0.002–0.220]	0.002
	SEA IgG4	49.667	[12.957–190.384]	0.000
***Sm*** **PCR^+^ versus uninfected**				
	age group			0.011
	young	10.727	[2.009–57.263]	0.005
	adolescent	5.228	[1.454–18.794]	0.011
	SEA IgG4	28.818	[6.700–123.964]	0.000
	SEA IgE	0.102	[0.011–0.960]	0.046
	SWA IgG3 × 100	1.418	[1.176–1.710]	0.000
**egg^+^ versus *Sm*PCR^+^**				
	not exposed	0.124	[0.042–0.367]	0.000
	SEA IgG4	3.095	[1.111–8.619]	0.031
	SEA IgE	12.212	[1.580–94.354]	0.016
	*Sm*CTF IgG1 × 100	0.802	[0.698–0.921]	0.002
	*Sm*CTF IgG2 × 100	1.150	[1.028–1.287]	0.015
	*Sm*CTF IgG3	0.000	[0.000–0.018]	0.001

## Data Availability

The data presented in this study are available on request from the corresponding author. The data are not publicly available due to ethical restrictions.

## Supplementary Materials

The following are available online at https://www.mdpi.com/article/10.3390/pathogens11010019/s1, Figure S1: Highest levels of *Sm*CTF-specific IgGs in adolescent individuals.
