# Supplementary material for: Distinct Schistosoma mansoni-Specific Immunoglobulin Subclasses Are Induced by Different Schistosoma mansoni Stages—A Tool to Decipher Schistosoma mansoni Infection Stages
Source: Pathogens. 2021 Dec 24;11(1):19. doi: 10.3390/pathogens11010019 (PMC8778779; doi:10.3390/pathogens11010019)
Supplement: Supplementary file 1 [file pathogens-11-00019-s001.zip › pathogens-1438015-supplementary.pdf]

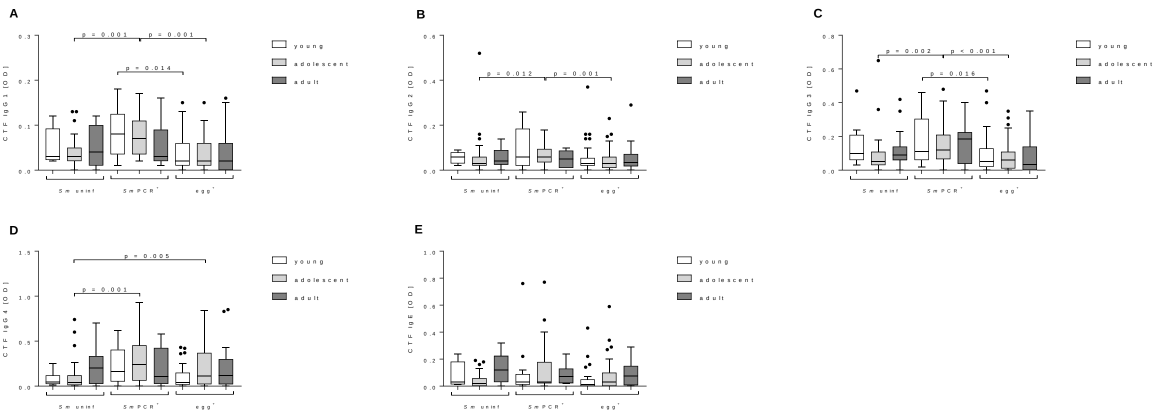

Supplementary Figure S1. Highest levels of *Sm*CTF-specific IgGs in adolescent individuals. Serum samples from all study participants (*Sm* uninfected, *Sm*PCR$^+$, egg$^+$) were analyzed for levels of *Sm*CTF-specific antibodies using ELISA technology. Each of these three groups was then split into the age groups "young" (4–9 years old, *Sm* uninfected n= 8, *Sm*PCR$^+$ n= 13, egg$^+$ n=33), "adolescent" (10–19 years old, *Sm* uninfected n= 31, *Sm*PCR$^+$ n= 41, egg$^+$ n=53) and "adult" (20–80 years old, *Sm* uninfected n= 21, *Sm*PCR$^+$ n= 8, egg$^+$ n=22). Graphs show the optical density of CTF-specific IgG1 (A), IgG2 (B), IgG3 (C), IgG4 (D) and IgE (E). Data are shown as box whiskers with median, interquartile ranges and outliers. Since data were non-parametric, statistical significances between the indicated groups were obtained after Kruskal-Wallis and Mann-Whitney-U tests within the different age groups as indicated by the brackets.
